# Need for Comprehensive Health Care Quality Measures for Older Adults

**DOI:** 10.1089/pop.2017.0109

**Published:** 2018-08-01

**Authors:** Stephanie MacLeod, Kay Schwebke, Kevin Hawkins, Joann Ruiz, Emma Hoo, Charlotte S. Yeh

**Affiliations:** ^1^ Advanced Analytics, Optum, Ann Arbor, Michigan.; ^2^ Quality Measurement, Optum, Eden Prairie, Minnesota.; ^3^ UnitedHealthcare Medicare & Retirement, Minneapolis, Minnesota.; ^4^ Pacific Business Group on Health, San Francisco, California.; ^5^ AARP Services, Inc., Washington, District of Columbia.

**Keywords:** quality measures, older adults, Medicare, health care expenditures, clinical practice guidelines, social determinants of health

## Abstract

Research indicates that older adults receive only about half of their recommended care, with varying quality and limited attention to social issues impacting their health through the most commonly used quality measures. Additionally, many existing measures neglect to address nonclinical social determinants of health. Evidence of the need for more comprehensive measures for seniors is growing. The primary purpose of this article, which is supported by a limited review of literature, is to describe gaps among current quality measures in addressing certain nonclinical needs of older adults, including key social determinants of health. In doing so, the authors describe their position on the need for expanded measures to incorporate these factors to improve care and quality of life. The authors conducted a limited review of the literature to inform this article, focusing specifically on selected measures for older adults rather than a broader systematic review of all measures. Most research identified was related to clinical practice guidelines rather than quality measures of care as applied to older adults. Furthermore, the literature reviewed reflected limited evidence of efforts to tailor quality measures for the unique social needs of older adults, confirming a potential gap in this area. A growing need exists for improved quality measures specifically designed to help providers address the unique social needs of older adults. Filling this gap will improve overall understanding of seniors and help them to achieve optimal health and successful aging.

## Introduction

In the United States, more than 46 million Americans are aged 65 years or older, a number that is increasing rapidly and expected to nearly double to approximately 83 million by 2050.^1–4^ Individuals aged 85 years and older represent the fastest growing segment of the population, with those aged 100+ the second fastest growing.^1,3,5^ Older adults often have multiple chronic conditions and subsequently higher health care needs and costs; many visit multiple providers and are at increased risk for fragmented care.^6^ One study of Medicare beneficiaries reported that most visit 2 primary care physicians and 5 specialists in 4 different practices.^6^ Additionally, 67% of Medicare beneficiaries have 2 or more chronic conditions, 50% have 3 or more, and 37% have 4 or more; thus, they face the challenges of complex care coordination.^6–8^ Furthermore, many are especially vulnerable because of low health literacy; 59% of adults aged 65 and older have only basic or below basic health literacy.^9–11^ These individuals may have difficulty understanding the risks and benefits of treatment options, asking questions, or advocating for themselves. For older adults, the consequences of suboptimal care are particularly important. Overall, older individuals are particularly vulnerable later in life because of their advancing age, frailty, mortality risk, functional limitations, and increased risk during surgical procedures, with potentially serious consequences.^12^

Quality measures are intended to quantify processes, systems, and patient outcomes associated with high-quality health care.^13^ They drive improvements by determining areas in need of better quality, identifying differences in care or outcomes among populations, and improving care coordination.^13^ Quality measures often are supported by clinical practice guidelines (CPGs), which direct efforts to improve care for individual conditions.^7^ However, CPGs do not always translate into rational measures that apply to specific segments of patients, such as older adults.^7^

Furthermore, CPGs often focus on only 1 condition; they fail to provide comprehensive guidance for delivering care to the growing population of aging seniors with multiple chronic conditions and declining physical and mental health. CPGs designed to manage these chronic conditions do not always apply to seniors as they are often based on studies that do not include older populations as participants^14^; thus, different research approaches specifically examining older populations are warranted to improve quality measures for them.

Perhaps more importantly, CPGs neglect to incorporate the nonclinical social, psychological, and environmental factors so critical to successful aging. These include not only the broader community-related social determinants of health, but also social “fabric of life” factors or determinants, such as the burden of self-management and care coordination, adherence with complex medication regimens, patient/caregiver preferences, caregiving for an ailing spouse or partner, rising costs of care, loneliness, social support, and purpose in life.^7,15^ These factors are difficult to assess, but are far more important to older adults than any one clinical condition.

The primary purpose of this article, which is supported by a limited yet thorough review of literature focusing on older adults, is to describe potential gaps and weaknesses in current health care quality measures to address the important personal and social concerns of older adults, including the social determinants of health and other related factors impacting quality of life. In doing so, the research team will describe their position on the need for more comprehensive, expanded measures designed to incorporate these unique factors impacting their health care, health outcomes, longevity, and overall quality of life in later years.

## Methods

To inform this paper and support the research team's position, a limited and specifically targeted review of the scientific literature was conducted in areas relevant to clinical and nonclinical quality measures specifically addressing the needs of older populations. The main goal was to provide an article on gaps in current quality measures in addressing the unique personal and social issues and concerns of older adults. The research team subsequently intends to support the growing need for more comprehensive measures incorporating the nonclinical issues impacting their lives. Thus, the search methods were restricted in order to meet that purpose, as this was not intended as a comprehensive and traditional systematic review of all literature published in this subject area.

Beginning in July of 2016, online search engines were utilized to identify research supporting the researchers' purpose and the perspectives discussed in this article. PubMed, Medline, Google Scholar, and a mainstream Google search were the resources utilized in the search; PubMed provided the majority of relevant research. Publications most closely aligned with the areas of interest were selected for inclusion in reviewing the results of the search in further detail. In some instances, references cited in relevant publications also were considered.

The following specific search terms and phrases were used to conduct the search: “quality measures for older adults,” “lack of quality measures for older adults,” “social determinants of health,” “social fabric,” “social concerns of older adults,” “priorities of older adults,” “evidence-based guidelines for older adults,” “clinical guidelines for older adults,” and “CMS PQRS measures.”

The initial assessment of results included a review of the titles of publications and, once relevant titles were examined, abstracts closely aligned with the search terms, primary purpose, older adult population, and perspective in this article. The primary selection criteria also included consideration of the publication date, with many of the chosen publications dated 2010 or later. Studies published in very recent years were prioritized, although those published more than 5 years ago also were considered and some chosen because of their relevancy in providing background content and support.

Elimination criteria included studies that were very general or broad in scope, and those detailing specific quality measurement tools/software or CPGs designed strictly for single conditions. Furthermore, the researchers primarily considered studies conducted in the United States, with a few exceptions of relevant research conducted elsewhere, as the main purpose focuses on current issues within the US health care system. Although the search was intended to focus primarily on quality measures, most research identified in this area was more specifically related to CPGs, confirming a gap in the literature and highlighting the need for attention to nonclinical issues among current quality measures for older adults.

Finally, once an initial pool of articles was selected, those publications were reviewed carefully in outlining and drafting this article. From the content of the research studies and reviews selected, areas of need or weakness among existing health care quality measures were assessed and identified by comparing the measures to the social determinants of health and other social concerns and quality of life issues considered important as subjects of this article. Thus, the researchers identified supporting research for the perspectives in this article with a streamlined review of the literature as described.

## Results

PubMed was the primary resource and provided the vast majority of references. The initial search for each term and phrase returned a number of results too large to examine individually. Thus, it was necessary to limit these results with the advanced search feature on PubMed, using the MeSH Terms filter. In this advanced search, the following numbers of publications were returned for each search term: quality measures for older adults: 3,798; lack of quality measures for older adults: 162; social determinants of health: 1,052; social fabric: 65; social concerns of older adults: 683; priorities of older adults: 565; evidence-based guidelines for older adults: 690; clinical guidelines for older adults: 2,788; and CMS PQRS measures: 14. These results were further narrowed by applying the selection and elimination criteria described previously in order to identify the most relevant publications. The final number of references ultimately included herein totals 42, with the vast majority published between 2010 and 2017. Several selected articles providing definitions, background information, or historical content published earlier were included as well.

### Summary of results

#### Examining current quality measures

Many quality measures currently used for Medicare-eligible individuals are based on the Centers for Medicare & Medicaid Services' (CMS) Five-Star Quality Rating System (STAR), intended to measure beneficiaries' quality of and experiences with care and to help them choose the best plans for their needs.^16,17^ However, the STAR system does not incorporate all factors that are important to older adults and as such is not specifically applicable to this population. In addition, financial incentives and industry pressure often influence providers and insurance companies to align their care and payments with STAR measures, even though exclusively using these measures alone, without consideration of other factors important in care management, is not always in the aging patient's best interests.

Many STAR measures are derived from the Healthcare Effectiveness Data and Information Set (HEDIS) developed by the National Committee for Quality Assurance, which provides quality measures for providers, plans, and health care organizations. Generally, HEDIS measures assess performance in specific aspects of care and address a range of single health issues.^18^ The latest set published in 2015 (with updates in 2016 and 2017) includes specific measures across 5 domains.^19^

Although HEDIS originated as an effort from employers and quality experts, the current measure set impacts more than the employed population. For example, several measures apply to the non-employed, including children/adolescents, Medicare (as part of an adult spectrum), and Medicaid.^18,19^ Several are intended to assess care for older adults; for instance, an effectiveness of care measure examines the use of influenza vaccinations for adults aged 65 and older. A fall risk management measure is also included; it is not designed for a specific age group but is useful in older adults' care management because many seniors are at high risk of falling. Similarly, HEDIS includes measures to evaluate the use of high-risk medications and potentially harmful drug–disease interactions in the elderly, as well as a Medicare Outcomes Survey measure.^19^ However, overall only a small number of these measures specifically target the elderly. In fact, 37 of the 86 specific measures (43%) do not apply to Medicare beneficiaries, and only 4 of them (less than 5%) focus primarily on social issues among older adults (ie, fall risk management, urinary incontinence, smoking and tobacco use cessation, mental health utilization). Thus, as with the STAR system, they do not comprehensively address the unique issues of older individuals.

In 2000, researchers at the RAND Health Corporation developed the Assessing Care of Vulnerable Elders (ACOVE) quality indicators, to evaluate care delivered to older Americans.^12,20^ ACOVE highlights 22 individual clinical conditions that account for most of the care seniors receive. For these conditions, RAND has established 236 quality indicators to set standards for care; the indicators encompass prevention, diagnosis, treatment, and follow-up.^12^ As with the HEDIS set, although the ACOVE indicators incorporate certain nonclinical issues (eg, suicide risk, driving ability, advance directives), they do not thoroughly address most of the unique social concerns and determinants of health impacting older adults and their quality of life.^21^

CMS also has established the Physician Quality Reporting System (PQRS), which primarily aims to allow and encourage providers and group practices to report information on quality of health care to Medicare. The system, which in 2017 transitioned to the similar Merit-Based Incentive Payment System, incorporates measures that focus on older adults, including issues not strictly related to clinical conditions and concerns.^22^ The specific PQRS measures do attempt to address some of the important personal and social aspects of health care, such as medication management, patient communication with physicians, appropriate care planning, risk of falls, elder maltreatment screening, tobacco use cessation, and assessment of sleep problems. However, many of the measures remain focused more on clinical conditions and aspects of care rather than on the social determinants of older adults' health and quality of life, and assessment of the measures has revealed other weaknesses as well.^23,24^ Furthermore, many providers and group practices have not consistently participated in reporting and continue to find it challenging to do so for various reasons.^24^ For one, research indicates that providers generally do not believe that the PQRS measures ensure high-quality care.^23^ In addition, low participation rates also may result from other factors including the costs and time burden of data collection, reporting, and submission; the complexity of the system and understanding the measures; the investment in technology required to participate; additional costs of implementation; eligibility thresholds; the potential for reporting errors; and the self-reported nature of the measures.^23,24^ Therefore, as with other quality measures, PQRS does not provide a thorough context for assessing the comprehensive nonclinical attention providers give to an aging population.

Finally, although efforts to develop nonclinical measures of quality have begun to emerge, this area is still lacking and often the social factors that impact older adults are not systematically addressed when care is provided. In the private sector, at least 1 large private health insurer has developed a “life situation” questionnaire to assess social determinants including a patient's living situation, financial and housing needs and shortfalls, food security, transportation access, activities of daily living, and other concerns.^25,26^ However, this type of questionnaire does not necessarily align with existing quality measures. In fact, although social determinants of health have been incorporated into the *International Classification of Diseases, Tenth Revision* coding system, there is currently no existing “crosswalk” in place to map these codes to other clinical condition codes or electronic health records. Thus, although private sector efforts are needed, much work remains to be done in this area to address older adults' quality of care.

#### Weaknesses of current quality measures in addressing care for older adults

Overall, measures derived from commonly accepted CPGs have been criticized for their lack of direct applicability to older adults and inability to translate into feasible quality measures, especially for those with multiple comorbidities.^27,28^ Current measures tend to support a disease-focused rather than patient-focused approach to care, and neglect to address the complexities of aging coupled with managing multiple chronic conditions.^7,18^ Many focus primarily on single common health conditions (eg, cancer, coronary artery disease, back pain, acute bronchitis, headache, stroke) and various services (eg, mammography, endoscopy, bone scans, cardiac stress testing, repeat imaging), but lack further specificity.^29^ Elsewhere, additional research demonstrates that CPGs do not provide an adequate framework for developing measures to assess quality of care for older adults while considering their unique needs, conditions, social situations, and personal preferences.^7^ In an analysis of 14 CPGs for chronic conditions common among the elderly (eg, diabetes, hypertension, heart failure, osteoporosis, stroke), researchers found that only 5 guidelines provided recommendations for frail adults aged 80 and older.^14^ In addition, fewer than 2% of studies examined reported a mean age of 80 years and older.

Existing quality measures also generally neglect to account for the rising average life expectancy and accelerating growth of the population aged 85 and older. Several HEDIS measures have age limits and are not used for adults over certain ages as determined by clinical guidelines (ie, 75 years), thus excluding the fastest growing populations of Americans.^7,20,30^ More importantly, although HEDIS does include clinical measures for older adults, the psychological, social, and other nonclinical aspects important to their quality of life are not thoroughly addressed. Furthermore, many of these clinical measures focus strictly on increased longevity and reduced morbidity, while for older adults, the quality of those longer years is just as important. Purpose in life, overall life satisfaction, resilience, and quality of life in later years with reduced burden on family members are critical aspects to consider, rather than just lifespan measured in terms of chronological age.

## Discussion

Research demonstrates a growing need to better balance older adults' priorities and nonclinical concerns with the importance of assessing clinical quality using existing measures. In various studies, the quality of life in later years has emerged as a key priority among seniors, even when compared with longevity. In one study surveying Medicare patients dealing with a terminal illness, researchers found that 86% would rather be at home than in a health care facility during their last 6 months of life.^31^ In addition, most participants reported they would prefer not to be on a ventilator in order to extend their lives and would consider drugs to improve their symptoms even if those drugs could shorten life.^31^ Elsewhere, a focus group survey asked frail elderly patients and their caregivers, “What is most important to you?” Overall, respondents' answers focused on “time spent at home” as a top priority.^32^ Similarly, a study examining the importance of various quality of life concerns among older adults across 22 countries found that participants ranked the ability to perform activities of daily living, autonomy (freedom and independence), mobility, happiness and life satisfaction, and social help/support among the most important to them.^33^ However, a gap still exists in the literature focusing strictly on quality of life issues of older adults. A review of 47 studies using quality of life assessments with older adults found that only 2 studies (4.2%) provided evidence of the personal importance given to quality of life within this population.^34^

Meanwhile, both health care policy and spending in the United States are dominated primarily by payment for medical treatments and services rather than prevention efforts and the nonmedical determinants of health including the quality of life issues described previously.^15,35^ Although estimated health care spending in the United States exceeds $3 trillion, the health outcomes of Americans continue to lag behind those of other industrialized countries,^15,36^ many of which dedicate more attention to social concerns such as loneliness, social isolation, and resilience. Estimates suggest that about 95% of US health care spending goes toward medical services, with only the remaining 5% to population health approaches to prevention, and improving overall health.^35^ However, although medical services are critical to disease management and optimal health, research indicates that clinical care is a weaker determinant of overall health compared to behavioral and other factors, such as diet and exercise.^15,37^

**FIG. 1.**
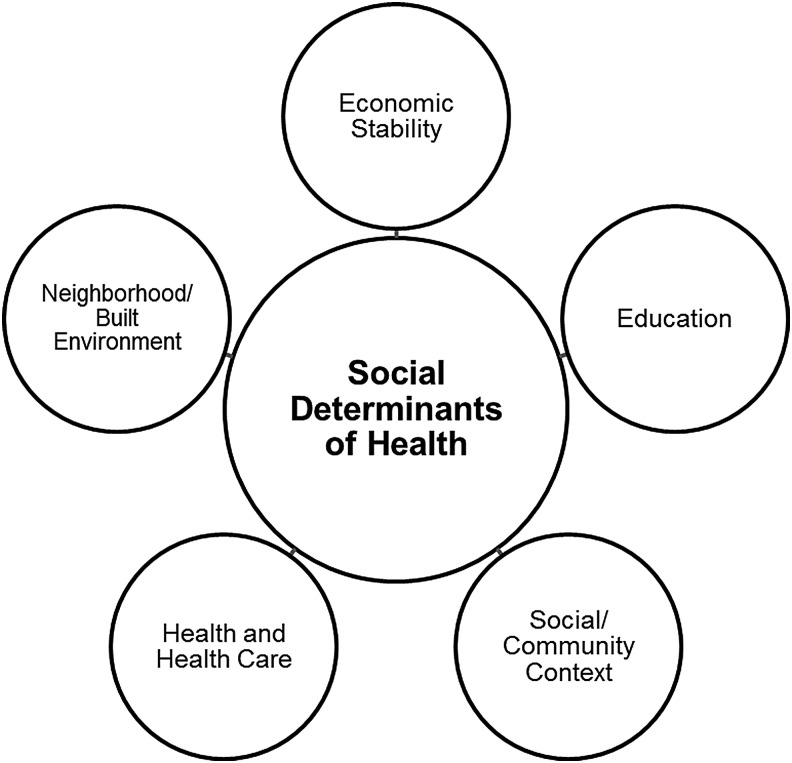
Five categories of the social determinants of health.^38^

Some of the most important influences often overlooked in care and specifically in quality measures include social circumstances, environmental conditions, psychological factors, and behaviors.^15,35^ Among the factors impacting overall health, key social, environmental, and behavioral factors account for 60% of influences, compared to 20% each attributed to medical care and genetics.^35^ These and other nonclinical influences are considered social determinants of health and have a significant impact on quality of life,^15^ especially among older adults. Social determinants of health (SDOH) are defined as the community- and population-related conditions in which people live, learn, work, and age that impact a range of health, functioning, and quality of life outcomes and risks.^38,39^ Healthy People 2020, an initiative of the US Department of Health and Human Services, recognize 5 key categories of SDOH: economic stability, education, social and community context, health and health care, and the neighborhood and built environment (Fig. 1).^38^ In addition to health care, the other 4 categories are equally important for older adults' quality of life, well-being, and overall health, yet issues such as finances, social context (ie, social support), and living environments are not considered to be part of commonly used health care quality measures.

Related to these common SDOH are the more individual, personal factors that also are considered components of social health for older adults. These include purpose in life, optimism, resilience, loneliness, social isolation, social support, caregiving, changes in routine, shifting social roles/retirement, loss of driving ability and physical mobility, loss of a spouse/loved one, living situations (eg, moving, being homebound), financial literacy, fixed incomes, life satisfaction, stereotypes of aging, and overall well-being. Among older adults, many of these social determinants are even more prevalent than common clinical conditions (eg, heart failure, stroke, diabetes, arthritis), and often are more impactful. For instance, high resilience later in life has been shown to lead to better health outcomes in older adult populations.^40^ Elsewhere, with regard to outcomes, loneliness among older adults has shown a larger negative impact on patient satisfaction with providers, health plans, and overall health care than any single medical condition.^41^ Finally, research confirms that homebound older adults have more chronic conditions and hospitalizations and are likely to be dissatisfied with their providers and health insurance.^42^

Overall, measuring quality of care for older adults presents unique challenges, as the risks and benefits of treatment decisions differ because of their higher prevalence of multiple comorbidities and complex medication regimens, leading to different management approaches.^14,20,27^ Care management for older adults also requires attention to common SDOH and other related social factors impacting their physical, mental, and social well-being, quality of life, and wide range of preferences.^20^ However, important concerns such as social support, social isolation, loneliness, resilience, and purpose in life are often overlooked in care provision for various reasons. In today's health care system, quality measures typically are designed to assess what providers actually do, rather than what patients want.^31^ Regardless of older patients' priorities, quality measures tend to focus strictly on clinical conditions rather than on their unique psychological and social needs. In addition, existing measures do not comprehensively examine the performance of providers in assessing the burden on older patients and their caregivers of coordinating various tests, treatments, appointments, and medications needed for the management of multiple conditions with multiple providers. The impact of this complex self-management of health needs is inadequately addressed by current measures, potentially impacting quality of life.

Finally, the lack of attention to nonclinical issues in assessing care delivered to older adults also has potential implications for health care utilization and expenditures because of the demonstrated health impacts of nonclinical social considerations. Considering not only the direct health and quality of life impacts but also the burden of care management, financial consequences, and other potential outcomes of inadequate care quality for older adults, the development of better measures specifically targeting this population is warranted.

### Limitations

The purpose of this article, supported by a streamlined, specific review of literature focusing on older adults, was to highlight the need for more comprehensive, expanded quality measures designed to address the nonclinical, social determinants of their health and quality of life. To meet this purpose, a restricted search methodology was used as the entire field of literature in the area of quality measures is extremely broad in scope. Although this approach may have limited the final results and selection of resources, the search was designed specifically to address the issues discussed within. Finally, examining every existing, individual quality measure applied in today's health care system would have been exhaustive and outside the scope of this article; thus, a minimal number of measures addressing older adults' social concerns may have been overlooked inadvertently.

### Future considerations

As research described here and elsewhere indicates, a growing need exists for broader quality measures to address the unique, nonclinical needs of older adults, including their SDOH and other social health-related concerns. Although clinical aspects of care remain the basis for quality measures, expanded measures for at-risk older adults ideally should incorporate not only these social, psychological, and environmental issues but also the burdens of care coordination and self-management of health, patient/caregiver literacy, and individual treatment preferences. Primarily, as the researchers have suggested, increased focus needs to be placed on the important SDOH impacting older adults' overall health and quality of life. This would require assessing key factors considered SDOH and additional personal social health issues including cognitive changes or decline, living arrangements, financial security, food security, independence, psychological losses (eg, retirement, death of a spouse or loved one), social support and connectedness, transportation, mobility, purpose in life, optimism, and resilience, among others. Furthermore, the inclusion of family members and/or caregivers in medical decisions should be a component of quality assessment of care delivered to older adults, as they can be critical in helping to facilitate care coordination and positive outcomes for patients. Greater attention to these concerns would help establish a more well-rounded approach to patient-centered care and potentially better quality, leading to improved outcomes among older adults.

## Conclusions

This article, supported by a targeted review of relevant literature, demonstrates the need for broader, more comprehensive health care quality measures to address the basic needs and social concerns of older adults. As the populations of adults ages 65+, 80+, and even 100+ continue to grow in numbers, quality of care assessment must consider the increasingly important nonclinical needs of these individuals to ensure optimal quality of life and health outcomes. As various health care stakeholders have begun to recognize, SDOH and related social health factors have the potential to significantly impact the lives and overall health of seniors just as much as clinical conditions, and thus should be incorporated into expanded quality measures. Development and implementation of measures specifically targeting these social, psychological, and environmental factors will be challenging. However, the potential positive implications include improved patient outcomes and satisfaction, higher quality of life in later years, potential reduced costs, and better quality of care along with increased efficiency in caring for older adults. As such, continued research to expand quality measures for this growing population is warranted.
